# Cytokine-Mediated STAT3 Transcription Supports ATGL/CGI-58-Dependent Adipocyte Lipolysis in Cancer Cachexia

**DOI:** 10.3389/fonc.2022.841758

**Published:** 2022-06-15

**Authors:** Aakash Y. Gandhi, Jinhai Yu, Arun Gupta, Tong Guo, Puneeth Iyengar, Rodney E. Infante

**Affiliations:** ^1^Center for Human Nutrition, University of Texas Southwestern Medical Center, Dallas, TX, United States; ^2^Harold C. Simmons Comprehensive Cancer Center, University of Texas Southwestern Medical Center, Dallas, TX, United States; ^3^Department of Molecular Genetics, University of Texas Southwestern Medical Center, Dallas, TX, United States; ^4^Department of Radiation Oncology, University of Texas Southwestern Medical Center, Dallas, TX, United States; ^5^Department of Internal Medicine, University of Texas Southwestern Medical Center, Dallas, TX, United States

**Keywords:** cancer cachexia, adipose wasting (malnutrition), adipocyte lipolysis, leukemia inhibitor factor, interleukin-6 (IL-6), adipose triglyceride lipase (ATGL), signal transducer and activator of transcription 3 (STAT3), β-adrenergic signaling

## Abstract

Adipose tissue inflammation is observed in multiple metabolically-altered states including cancer-associated cachexia and obesity. Although cachexia is a syndrome of adipose loss and obesity is a disease of adipose excess, both pathologies demonstrate increases in circulating levels of IL-6 family cytokines, β-adrenergic signaling, and adipocyte lipolysis. While β-adrenergic-stimulated adipocyte lipolysis is well described, there is limited mechanistic insight into how cancer cachexia-associated inflammatory cytokines contribute to adipocyte lipolysis under pathologic conditions. Here, we set out to compare adipocyte lipolysis signaling by cancer cachexia-associated IL-6 family cytokines (IL-6 and LIF) to that of the β-adrenergic agonist isoproterenol. Unlike isoproterenol, the IL-6 family of cytokines required JAK/STAT3-dependent transcriptional changes to induce adipocyte lipolysis. Furthermore, cachexia-associated cytokines that used STAT3 to induce lipolysis were primarily dependent on the lipase ATGL and its cofactor CGI-58 rather than lipases HSL and MAGL. Finally, administration of JAK but not β-adrenergic inhibitors suppressed adipose STAT3 phosphorylation and associated adipose wasting in a murine model of cancer cachexia characterized by increased systemic IL-6 family cytokine levels. Combined, our results demonstrate how the IL-6 family of cytokines diverge from β-adrenergic signals by employing JAK/STAT3-driven transcriptional changes to promote adipocyte ATGL/CGI-58-dependent lipolysis contributing to adipose wasting in cancer cachexia.

## Introduction

Inflammation of adipose tissue occurs during phenotypic extremes of adiposity. Cachexia, a syndrome of chronic inflammation defined by rapid losses in weight, adipose, and muscle, is associated with net decreases in adipose triglyceride content concomitant with increases in systemic and local tissue (adipose) levels of inflammatory cytokines (1–3). Cachexia is observed in 50% of all solid tumor patients, responsible for 30% of all cancer-related deaths, and ultimately an independent risk factor for reduced cancer-specific mortality (1, 4). Obesity, a disease of adipose excess, represents the opposite end of the metabolic spectrum from cachexia with net increases in adipose triglyceride content while still associated with similar cytokine profiles and signaling events in adipose tissue (5–7). Obesity is a significant risk factor in the development of metabolic syndrome and cancer development, affecting morbidity and mortality in 1/3 of the world’s population. Although inflammation is independently associated with both metabolic conditions, there is limited mechanistic insight into how common inflammatory cells and cytokines facilitate the mobilization of lipids from adipose tissue to regulate cachexia and obesity development.

Though adipose is composed of many cell types, almost all adipose fat is sequestered into adipocyte lipid droplets as the neutral storage form triacylglycerol (TAG) (8). Adipocyte lipolysis is an enzymatic process by which TAG is sequentially hydrolyzed into glycerol and non-esterified fatty acids (NEFA) by adipose triglyceride lipase (gene: *Pnpla2*, protein: ATGL) and its cofactor CGI-58 (gene: *Abhd5*, protein: CGI-58), hormone sensitive lipase (gene: *Lipe*, protein: HSL), and monoacylglycerol lipase (gene: *Mgll*, protein: MAGL) (8). The catecholamine-mediated β-adrenergic signaling pathway induces TAG breakdown through G protein-coupled receptor-dependent activation of adenylate cyclase producing cAMP, which in turn activates protein kinase A and its subsequent phosphorylation of multiple targets (e.g. HSL and perilipin proteins) to increase lipolysis (9, 10). Like cytokine-associated inflammation, increased β-adrenergic signaling of host adipose has been associated with cachexia development (11–13). However, unlike in catecholamine-induced signaling, the mechanisms of cancer cachexia-associated TAG lipolysis by IL-6 family inflammatory cytokines are unknown.

We and others have shown that murine cancer cachexia models have elevated serum levels of IL-6 and LIF, which contribute to adipose wasting in this syndrome (2, 14–18). This family of cytokines activates Janus kinase (JAK) signaling pathways in a number of cell types, including adipocytes (15, 19, 20). We previously demonstrated that inhibition of JAK suppresses IL-6 family cytokine adipocyte signaling and lipolysis and blocked cachexia-associated adipose wasting and anorexia in the C26c20 murine cancer cachexia model (15). In this manuscript, we set out to further characterize the role of IL-6 family cytokine-induced inflammation on adipocyte lipolysis. We used pharmacologic and reverse genetic approaches to compare lipolysis signaling between the IL-6 family of cytokines and the classical β-adrenergic agonist isoproterenol in differentiated adipocytes. Our data identified differences in the mechanisms that cancer cachexia-associated cytokines and isoproterenol used to induce lipolysis. Unlike isoproterenol, IL-6- and LIF-induced adipocyte lipolysis was dependent on JAK signaling, STAT3 activation, and *de novo* transcriptional/translational changes in adipocytes. In contrast, isoproterenol-induced lipolysis could be blocked only by the β-adrenergic inhibitor propranolol. These results were further supported by *in vivo* with findings demonstrating that cancer cachexia-induced adipose loss could be suppressed by JAK inhibition but not propranolol. Further downstream, the IL-6 family cytokines specifically induced lipolysis through the actions of the lipase ATGL and its cofactor CGI-58, but did not require the lipases HSL and MAGL. Altogether, these findings highlight a mechanism by which cancer cachexia-associated IL-6 family cytokines induce ATGL/CGI-58-dependent lipolysis and adipose wasting through JAK/STAT3 signaling and subsequent *de novo* transcription/translation, diverging significantly from the traditional sympathetic β-adrenergic induction of adipocyte lipolysis. Further understanding of these differences of adipocyte liplolysis induction could provide novel therapeutic targets for patients suffering from cancer cachexia.

## Methods

### Buffers and Culture Medium

The identity and sources of key commercially-obtained reagents used in this study are described in **Table 1**. Buffer A contained 10 mM Tris-HCl (pH 6.8), 100 mM NaCl, 1% (w/v) SDS, 1 mM EDTA, 1 mM EGTA, phosphatase inhibitor cocktails Set I and Set II (1:1,000 v/v), and protease inhibitor cocktail (1:1,000 v/v). Medium A was DMEM high glucose supplemented with 100 U/ml penicillin and 100 µg/ml of streptomycin sulfate. Medium B was Medium A supplemented with 10% (v/v) FCS. Medium C was Medium A supplemented with 5% (v/v) FCS. Medium D was DMEM without glucose, sodium pyruvate, glutamine, or phenol red supplemented with 10 mM glucose, 100 U/ml penicillin, 100 µg/ml streptomycin sulfate, and 0.4% (w/v) fatty acid-free bovine serum albumin (BSA).

**Table 1:** Key Resources.

REAGENT	SOURCE	IDENTIFIER
**Antibodies**		
Mouse monoclonal anti-β-Actin	Cell Signaling Technology	Cat# 3700
Mouse monoclonal anti-STAT3	Cell Signaling Technology	Cat# 9139
Mouse monoclonal anti-pSTAT3^Tyr705^	Cell Signaling Technology	Cat# 9138
Rabbit monoclonal anti-ATGL	Cell Signaling Technology	Cat# 2439
Rabbit monoclonal anti-HSL	Cell Signaling Technology	Cat# 18381
Rabbit monoclonal anti-pHSL^S660^	Cell Signaling Technology	Cat# 4126
Rabbit monoclonal anti-Plin1	Cell Signaling Technology	Cat# 9349
Mouse monoclonal anti-CGI-58	Novus Biologicals	Cat# H00051099-M01
Mouse monoclonal anti-MGLL	Santa Cruz Biotechnology	Cat# sc-398942
Peroxidase AffiniPure Donkey Anti-Mouse IgG	Jackson ImmunoResearch	Cat# 715-035-150
Peroxidase AffiniPure Goat Anti-Rabbit IgG	Jackson ImmunoResearch	Cat# 111-035-003
Can Get Signal Solution I	TOYOBO	Cat# NKB-201
**Chemicals and Recombinant Proteins**		
Precision Plus Protein Kaleidoscope Standards	BioRad	Cat# 1610375
Protease inhibitor cocktail	Calbiochem	Cat# 539131
Phosphatase inhibitor cocktail Set I	MilliporeSigma	Cat# 524624
Phosphatase inhibitor cocktail Set II	MilliporeSigma	Cat# 524625
Polybrene	MilliporeSigma	Cat# TR-1003-G
X-tremeGENE HP DNA Transfection Reagent	Roche	Cat# 6366236001
Insulin Solution	Cayman	Cat# 10008979
Dexamethasone	MilliporeSigma	Cat# D4902
Rosiglitazone	MilliporeSigma	Cat# R2408-50MG
IBMX (3-isobutyl-1-methylxanthine)	MilliporeSigma	Cat# I5879-5G
DMEM, no glucose, no glutamine, no phenol red	ThermoFisher	Cat# A1443001
Opti-MEM	ThermoFisher	Cat# 11058021
DMEM with high glucose	MilliporeSigma	Cat# D6429
Dulbecco’s Phosphate Buffered Saline (DPBS)	MilliporeSigma	Cat# D8537
Glucose	MilliporeSigma	Cat# G7021-100G
Glutamine	Corning	Cat# 25-005-CI
Bovine serum albumin (BSA), fatty-acid free	MilliporeSigma	Cat# A7030
Penicillin-Streptomycin Solution	ThermoFisher	Cat# 15140122
Fetal Bovine Serum (FCS)	Corning	Cat# 35-015-CV
Millex-HV Syringe Filter Unit, 0.45 µm, PVDF, 33 mm	MilliporeSigma	Cat# SLHV033RB
SuperSignal^TM^ West Pico PLUS Chemiluminescent Substrate	ThermoFisher	Cat# 34580
**Critical Commercial Assays**		
MycoAlert® Mycoplasma Detection Kit	Lonza	Cat# LT07-701
BCA protein assay Kit	ThermoFisher	Cat# 23225
NEFA-HR Color Reagent A	Wako Diagnostics	Cat# 999-34691
NEFA-HR Solvent A	Wako Diagnostics	Cat# 995-34791
NEFA-HR Color Reagent B	Wako Diagnostics	Cat# 991-34891
NEFA-HR Solvent B	Wako Diagnostics	Cat# 993-35191
NEFA-HR NEFA Standard Solution	Wako Diagnostics	Cat# 276-76491
Glycerol Standard Solution	MilliporeSigma	Cat# G7793
Free Glycerol Reagent	MilliporeSigma	Cat# F6428
**Inhibitors**		
Ruxolitinib	Selleckhem	Cat# INCB018424
Propranolol hydrochloride	MilliporeSigma	Cat# P0884

### Cell Culture

Stock cultures of mouse pre-adipocytes were derived from the adipose inguinal stromal vascular fraction fibroblasts as previously described (7). Pre-adipocytes, HEK293T, and RM9 cells were maintained in monolayer culture at 37°C in either medium B at 10% CO_2_ (pre-adipocytes) or 5% CO_2_ (HEK293T, RM9). Pre-adipocyte and HEK293T cells were passaged no longer than 3 weeks to minimize genomic instability. The cells were also tested for *Mycoplasma* contamination every six months using a MycoAlert *Mycoplasma* Detection Kit. Pre-adipocytes were differentiated to mature adipocytes as previously described (7). Briefly, pre-adipocytes were set up in a 12-well format on day -2 in 1 ml medium B at a density of 1 x 10^5^ cells/well. On day 0, medium was removed and differentiation was initiated with 1 ml medium B supplemented with 0.5 mM 3-isobutyl-1-methylxanthine, 1 µM dexamethasone, 10 µg/ml insulin, and 1 µM rosiglitazone. On day 2, the medium was removed and replaced with 1 ml medium B supplemented with 10 µg/ml insulin. On day 4 and 6, the medium was removed and replace with 1 ml of medium B. Differentiated adipocytes were used for experimentation on days 7 or 8.

### Lentiviral Production

Single guide RNA (sgRNA) targeting the indicated genes cloned into the *Bsm*BI site of the lentiCRISPRv2 vector (AddGene #52961) as previously described (21). The annealed oligonucleotides containing the *Bsm*BI compatible ends and the sgRNA target sequence incorporated into each sgRNA-lentiCRISPR/Cas9 construct as described in **Table 2**.

**Table 2:** Oligonucleotide Sequences for sgRNA-lentiCRISPR/Cas9 Constructs

Target Gene	Forward Sequence (5' to 3')	Reverse Sequence (5' to 3')
*GFP*	CACCGGTGAACCGCATCGAGCTGA	AAACTCAGCTCGATGCGGTTCACC
*Stat3(*1)	CACCGAGGCCTCAAGATTGACCTAG	AAACCTAGGTCAATCTTGAGGCCTC
*Stat3(*2)	CACCGAACATGGAGGAGTCTAACAA	AAACTTGTTAGACTCCTCCATGTTC
*Pnpla2*	CACCGAGAGGCGGTAGAGATTGCGA	AAACTCGCAATCTCTACCGCCTCTC
*Lipe*	CACCGAGTATGTCACGCTACACAA	AAACTTGTGTAGCGTGACATACTC
*Abhd5*	CACCGAGTTTGGATCAGGGCCCTAG	AAACCTAGGGCCCTGATCCAAACTC
*Mgll*	CACCGCTGTCTCGGAACAAGTCGG	AAACCCGACTTGTTCCGAGACAGC

For lentiviral production, HEK293T cells were plated at 1 x 10^6^ cells/100 mm dish in 10 ml medium B on day 0. On day 2 (~70-80% confluence), medium was then removed and replaced with 8 ml medium B supplemented with an additional 2 mM glutamine and the transfection cocktail: 15 µl X-tremeGENE HP DNA Transfection Reagent, 2.5 µg sgRNA-lentiCRISPRv2 (targeted to the indicated gene), 1 µg pMD2.G (AddGene #12259), and 1.5 µg psPAX2 (AddGene #12260). On day 3, medium was removed and treated with 11 ml medium A with 30% FCS supplemented with an additional 2 mM glutamine. On day 4, 5, and 6, virus-containing medium was collected and exchanged with 11 ml of fresh medium A with 30% FCS supplemented with an additional 2 mM glutamine. The collected virus-containing medium was filtered through a 0.45 µm syringe filter unit and stored at -80°C for future use.

### Generation of Knockout Pre-Adipocytes Lines Using CRISPR/Cas9

Stable genetically-ablated *GFP* (control), *Pnpla2* and *Lipe* pre-adipocytes were created as follows: On day 0, parental pre-adipocytes were plated at 1 x 10^5^ cells/100 mm dish in 10 ml medium B. On day 1, medium was removed and replaced with 2.5 ml medium B and 2.5 ml of the virus-containing medium derived from the indicated sgRNA-lentiCRISPR/Cas9 construct (described above), and polybrene was added to a final concentration of 8 μg/ml. A parental pre-adipocyte (non-infected) line was also maintained using 5 ml medium B with 8 μg/ml polybrene. On day 2, the medium was replaced with 10 ml medium B. On day 3, the medium was replaced with 10 ml of fresh medium B containing 3 µg/ml puromycin and subsequently replaced every two days until complete cell death was observed in the parental (non-infected) control line (~5 days). Additionally, during this selection period, virally-infected cells were split if their confluence was observed to be more than 50%. Following selection, cells were trypsinized and either alliquoted for cryopreservation or immediately passaged for differentiation.

Genetically-ablated *GFP* (control), *Stat3, Abhd5*, *Mgll* pre-adipocytes were created as follows: On day -2, parental pre-adipocytes were plated in a 12-well format at 1 x 10^5^ cells/well in 1 ml medium B. On day -1, medium was removed and replaced with 0.3 ml medium B and 0.2 ml of the virus-containing medium derived from the indicated sgRNA-lentiCRISPR/Cas9 construct (described above), and polybrene was added to a final concentration of 8 μg/ml. On day 0, medium was removed and the pre-adipocytes were continued on the standard adipocyte differentiation protocol as described above.

### NEFA and Glycerol Release Assays

Differentiated adipocytes were treated as indicated in the figure legends with commercial reagents (see **Table 1**) except for recombinant LIF which was expressed and purified as previously described (14). Medium was subsequently removed and NEFA and glycerol concentrations were measured as previously described (7, 14).

### Mouse Studies

Male wild-type C57BL/6J mice were obtained from Jackson Laboratories at ~8 weeks of age. All mice were allowed to acclimate in UT Southwestern animal facilities before experimentation for at least 1 week. Animals were housed 3-4 mice per cage, kept in a temperature-controlled facility with a 12 h light/dark cycle, and provided water and normal chow diet ad libitum. Approximately 200 g of standard chow diet was placed in each cage. When food reached ~100 g per cage, it was replenished to ~100 g. Food was weighed (digital Ohaus scale) at the same time daily and compared with the previous day’s weight to calculate the 24 h food intake per cage. This food intake was converted to energy density (kcal/g) and normalized per mouse body weight. Body weight was measured using a standard balance (digital Ohaus scale). Adipose tissue mass and lean tissue mass were measured longitudinally using ECHO MRI (ECHO Medical Systems) at 10 a.m. to noon at the indicated time points. For tumor studies, RM9 cells were plated 1 × 10^6^ cells/100 mm dish in 10 ml medium C. These cells were split/propagated and medium was replaced with fresh 10 ml of medium every 2 days until there was adequate amount of cells for each experiment. At day 0, 1 × 10^6^ RM9 cells in 100 μl of PBS were injected into the right hind flank of mice at day 0, and tumor volume was calculated by taking half of the product of the caliper (VWR) measurements of length, width, and breadth at the indicated time points. At the end of the experiments, mice were euthanized within 12 h of expected death in the tumor-bearing mice as recommended by the Institutional Animal Care and Use Committee by using a CO_2_ chamber, and organs were collected and snap frozen.

### Immunoblot Analysis

For *in vitro* differentiated adipocyte experiments, medium was removed after the indicated treatment and each well was washed twice with 1 ml PBS followed by addition of 150 µl buffer A. The 12-well plate was shaken using a titer plate shaker (Lab-Line Instruments) at room temperature for 5 min followed by transfer of the cell lysate into a 1.5-ml microcentrifuge tube. The recovered lysate was briefly subjected to centrifugation at 20,000 x *g* for 1 min at 4°C, followed by 10 s of sonication at 50% amplitude (Sonics VCX130 ultrasonic liquid processor, CV18 converter, 2 mm probe). The post-sonication lysate was subjected to centrifugation at 20,000 x *g* for 10 min, after which the supernatant was collected avoiding the lipid layer, and protein concentration was measured using the bicinchoninic acid kit (ThermoFisher) per the manufacturer’s directions. For mouse studies, tissues were processed for immunoblot analysis as previously described (14).

Cell culture or animal tissue extracts were mixed with 5X SDS loading buffer, heated at 95°C for 10 min, and 8 µg lysates were subjected to 11% SDS/PAGE, except for immunoblot analysis of pSTAT3^Tyr705^ and pHSL^Ser660^ which were subjected to 4-15% gradient SDS/PAGE (BioRad 4561086). The electrophoresed proteins were transferred to nitrocellulose membranes using the Bio-Rad Trans Blot Turbo system and followed by immunoblot staining with the following primary antibodies: IgG-β-actin (1:20,000), IgG-ATGL (1:1,000), IgG-CGI-58 (1:2000), IgG-HSL (1:1,000), IgG-pHSL^Ser660^ (1:1,000), IgG-MAGL (1:2,000) IgG-Plin1 (1:1,000), IgG-STAT3 (1:1,000), and IgG-pSTAT3^Tyr705^ (1:1,000). Bound antibodies were visualized by chemiluminescence (Super Signal Substrate, Pierce) by using a 1:10,000 dilution of donkey anti-mouse IgG (Jackson ImmunoResearch) or 1:5,000 of donkey anti-rabbit IgG (Jackson ImmunoResearch) conjugated to horseradish peroxidase. Membranes were scanned using an Odyssey FC Imager (Dual-Mode Imaging System, 2-minute integration time) and analyzed using Image Studio version 5.0 (LI-COR). Protein molecular weights were marked using Kaleidoscope Precision Plus Protein™ Standards (Bio-Rad).

### Real-Time PCR Analysis of Gene Expression

Extraction of total RNA with RNA-STAT-60 reagent from tumors and subsequent quantitative reverse transcriptase PCR assays for mRNA levels the indicated gene products were conducted as previously described (15). The sequences of primers (obtained from Integrated DNA Technologies) used in these studies are as described in **Table 3**.

**Table 3:** Oligonucleotide Sequences for Real-time PCR Analysis of Gene Expression

Target Gene	Forward Sequence (5' to 3')	Reverse Sequence (5' to 3')
*ActB*	CCGTGAAAAGATGACCCAGATC	CACAGCCTGGATGGCTACGT
*IL6*	TCGTGGAAATGAGAAAAGAGTTG	AGTGCATCATCGTTGTTCATACA
*IL6ra*	CCTGCCAGGGGCCACCGTTAC	TGTGAGCCAGAGTACACCCAGTGAAT
*Socs3*	CACCTGGACTCCTATGAGAAAGTG	GAGCATCATACTGATCCAGGAACT
*Lif*	AGCCGTTTCCCAACAACGT	CCGTTGCCATGGAAAGAT
*Lep*	CTCCATCTGCTGGCCTTCTC	CATCCAGGCTCTCTGGCTTCT

### Statistical Analysis and Graphs

Details of statistical analysis and graphs for each experiment can be found in the respective figure legend. Data are presented as mean ± SEM, dot plots ± SEM, or dot plots with bars ± SEM. For all graphs, curves were illustrated manually to guide the eye, and do not represent a best-fit. All statistical analyses were conducted in Prism 9 (GraphPad).

## Results

### Comparison of IL-6 Family Cytokine and β-Adrenergic Agonist Adipocyte Signaling and Lipolysis

**Figure 1:**
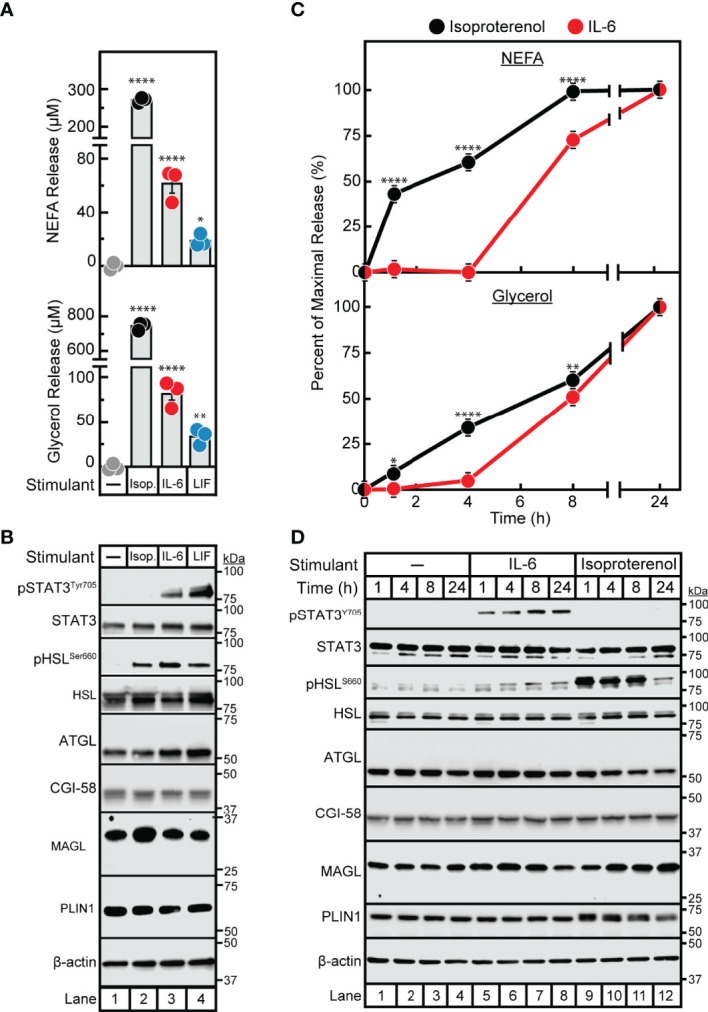
Comparison of IL-6 Family Cytokine and β-adrenergic Adipocyte Signaling and Lipolysis. **(A–D)** Differentiated adipocytes in a 12-well format were treated in a final volume of 1 ml of medium D with 3 µl DMSO and 3 µl PBS in the absence or presence of 300 nM isoproterenol (*black, Isop.*), 3 nM IL-6 (*red*), or 0.3 nM LIF (*blue*). After incubation (37°C, 10% CO_2_) for either 24 hours **(A, B)** or the indicated time **(C, D)**, medium was collected and processed to measure concentrations of NEFA (**A, C**; top panels) or glycerol (**A,C**; bottom panels), and adipocytes were harvested and processed for immunoblot analysis **(B, D)** with the indicated antibody as described in *Methods*. Data is shown as mean ± SEM of three wells. *p < 0.05, **p < 0.01, or ****p < 0.0001 based on one-way ANOVA followed by Dunnett’s multiple comparison test to compare each condition to the vehicle condition **(A)** or two-way ANOVA followed by Šidák’s multiple comparison test to compare isoproterenol to IL-6 conditions at matched time points **(B)**. These results were confirmed in at least three independent experiments.

Despite the association of cytokines with adipose inflammation in cachexia and obesity, their role in adipocyte lipolysis is not well-established. To begin understanding cytokine-induced adipocyte lipolysis, we initially compared adipocyte lipolysis by the cancer cachexia-associated cytokines IL-6 and LIF to that of the well-characterized lipolytic factor and β-adrenergic agonist isoproterenol. In **Figure 1A**, we treated differentiated adipocytes with isoproterenol or recombinant forms of IL-6 or LIF and evaluated the release of triglyceride lipolysis products non-esterified fatty acids (NEFA) and glycerol from adipocytes into the medium. Both NEFA and glycerol release are well recognized surrogates for levels of adipocyte lipolysis (22). We observed that IL-6, LIF, and isoproterenol all induced adipocyte lipolysis compared to vehicle treatment, though isoproterenol stimulated ~5- to 6-fold more NEFA release than IL-6 and approximately 10-fold more NEFA release than LIF (**Figure 1A**). All three treatments of adipocytes also induced glycerol release; isoproterenol-stimulated adipocytes had an ~10-fold and ~25-fold increase in glycerol release when compared to IL-6-stimulated adipocytes or LIF-stimulated adipocytes, respectively.

We next conducted immunoblot analysis of lysates from IL-6-, LIF-, and isoproterenol-treated adipocytes to compare adipocyte signaling capacity between the three lipolytic agents. As we have previously described, IL-6 and LIF but not isoproterenol activated the JAK/STAT3 pathway as demonstrated by increased phosphorylation of the Tyr705 residue of STAT3 (**Figure 1B**) (20). All treatments stimulated the activation of HSL in association with lipolysis induction as judged by increased phosphorylation of the Ser660 residue of HSL (9, 10). Importantly, neither isoproterenol, IL-6, nor LIF stimulated changes in levels of other lipolysis- or lipid droplet-associated proteins including total HSL, ATGL, CGI-58, MAGL and perilipin. Altogether, both IL-6 family of cytokine- and β-adrenergic agonist-stimulated adipocytes displayed an association between lipolysis induction and HSL phosphorylation, whereas only the cytokine-stimulated adipocytes demonstrated STAT3 phosphorylation with this lipolysis.

In parallel, we performed a time course assay to evaluate the kinetics of IL-6- and isoproterenol- induced maximal NEFA and glycerol release from adipocytes (**Figure 1C**). This was correlated with the timing of HSL and STAT3 activation as judged by immunoblot analysis (**Figure 1D**). Isoproterenol induced significant NEFA and glycerol release from adipocytes between 0-8 h, which was correlated with high levels of HSL phosphorylation in the absence of STAT3 phosphorylation. At the 24 h time-point, isoproterenol-induced adipocytes did not demonstrate further NEFA release, corresponding to significantly reduced HSL phosphorylation. IL-6-induced adipocytes did not have measurable NEFA or glycerol release until 8 h of incubation which was correlated with a minimal increase in HSL phosphorylation. STAT3 phosphorylation was induced by IL-6 as early as 1 h and remained phosphorylated through 24 h of incubation in these IL-6-treated adipocytes. None of the lipolytic stimulants reduced the expression of other lipolytic proteins (total HSL, ATGL, CGI-58, MAGL, or PLIN1) as judged by immunoblot analysis. Unlike isoproterenol which induced lipolysis immediately, cytokine-mediated lipolysis was delayed for ~8 h despite induction of early STAT3 phosphorylation.

### IL-6- but Not β-Adrenergic-Mediated Adipocyte Lipolysis Requires *De Novo* Transcription

**Figure 2:**
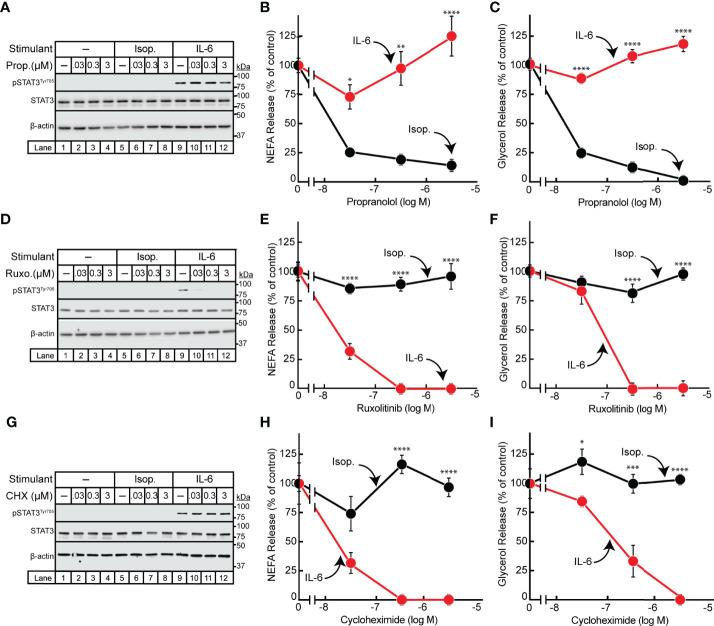
Effect of Inhibitors on IL-6- and β-adrenergic-treated Adipocytes. **(A–I)** Differentiated adipocytes in a 12-well format were treated in a final volume of 1 ml medium D with 6 µl DMSO and 3 µl PBS containing either 10 nM isoproterenol (*black, Isop.*) or 3 nM IL-6 (*red*) in the absence or presence of the indicated concentration of propranolol (**A–C**, *Prop.*), ruxolitinib (**D–F**, *Ruxo.*), or cycloheximide (**G–I**, *CHX*). After incubation for 24 hours, medium was collected and processed to measure concentrations of NEFA **(B, E, H)** or glycerol **(C, F, I)** and adipocytes were harvested and processed for immunoblot analysis **(A, D, G)** with the indicated antibodies as described in *Methods*. Data is shown as mean ± SEM of three wells. *p < 0.05, **p < 0.01, ***p < 0.001, or ****p < 0.0001 based on two-way ANOVA followed by Šidák’s multiple comparison test to compare isoproterenol to IL-6 conditions at matched time points. These results were confirmed in at least three independent experiments.

Having demonstrated differences in the timing of isoproterenol- and IL-6-induced adipocyte lipolysis, we next pursued identification of other points of mechanistic divergence between these two lipolytic stimulants. It is well established that β-adrenergic induction of lipolysis is inhibited by the pan β-blocker propranolol (23, 24). We have also previously shown that JAK inhibition suppresses IL-6 family cytokine-induced lipolysis in adipocytes (15). To establish that the IL-6 family of cytokines do not signal through the β-adrenergic lipolytic signaling pathway, we treated IL-6- and isoproterenol-stimulated differentiated adipocytes with increasing concentrations of either propranolol (**Figures 2A–C**) or the JAK inhibitor ruxolitinib (**Figures 2D–F**). As shown above, STAT3 phosphorylation was induced in the presence of IL-6 but not isoproterenol (**Figures 2A, D**). Increasing concentrations of ruxolitinib (**Figure 2D**), but not propranolol (**Figure 2A**), was able to block IL-6-mediated adipocyte STAT3 phosphorylation. Furthermore, increasing concentrations of propranolol blocked isoproterenol- but not IL-6-stimulated NEFA (**Figure 2B**) and glycerol (**Figure 2C**) release from adipocytes. Conversely, ruxolitinib blocked IL-6- but not isoproterenol-stimulated NEFA (**Figure 2E**) and glycerol (**Figure 2F**) release from adipocytes.

Since the IL-6 family of cytokine induction of JAK-dependent lipolysis was associated with the phosphorylation and activation of the known transcription factor STAT3, we next evaluated if IL-6-induced adipocyte lipolysis is dependent on *de novo* transcription/translation. IL-6- or isoproterenol-treated adipocytes were incubated with increasing concentrations of the translation inhibitor cycloheximide (**Figures 2G–I**). Cycloheximide had no effect on isoproterenol-induced adipocyte NEFA (**Figure 2H**) or glycerol (**Figure 2I**) release from adipocytes. However, cycloheximide suppressed the release of NEFA (**Figure 2H**) and glycerol (**Figure 2I**) release from IL-6-stimulated adipocytes without influencing STAT3 phosphorylation as demonstrated by immunoblot analysis (**Figure 2G**). Taken together, the IL-6 family of cytokines induce a JAK-dependent, β-adrenergic-independent lipolysis that is associated with STAT3 activation and requires *de novo* transcription/translation.

### Adipose Wasting in a Murine Cancer Cachexia Model Is Suppressed by JAK Inhibition but Not β-Adrenergic Inhibition

**Figure 3:**
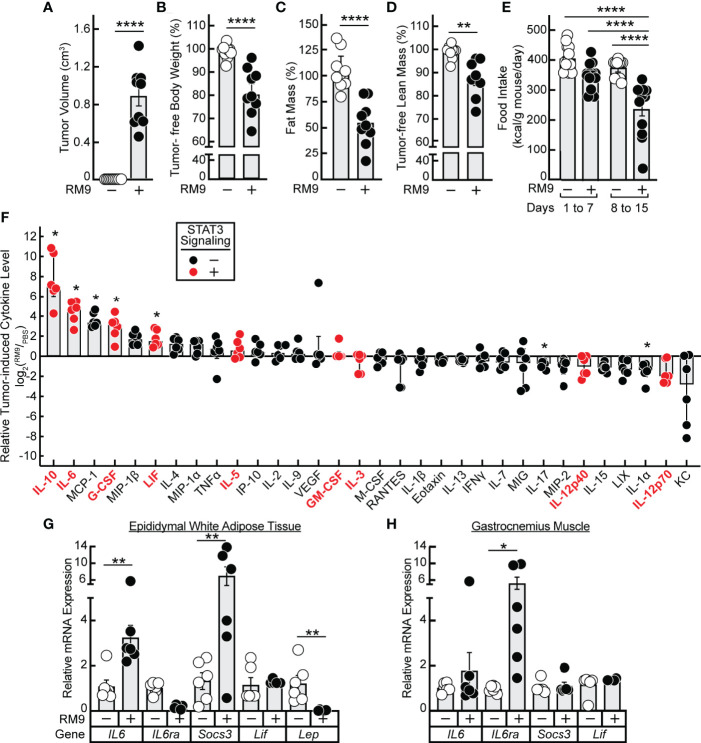
Serum Cytokine Profile of RM9 Cancer Cachexia Murine Model. **(A–H)** Chow-fed C57BL/6J mice (12-week old male, n=9) were injected s.c. in their right flank with 100 μl PBS in the absence (*open circles*) or presence (*closed circles)* of 1x10^6^ RM9 cells on day 0 with mice subsequently observed for 12 days. One mouse in the RM9 cohort died unexpectedly and the values of this mouse was omitted from the data. Measurements of tumor volume **(A)**, tumor-free body weight **(B)**, fat mass **(C)**, tumor-free lean mass **(D)**, and food intake **(E)** were obtained at sacrifice. Tumor-free body weight, fat mass, and tumor-free lean mass are shown relative to the average measurements of the mouse cohort that received PBS alone which were which were 27.2 g, 3.2g, and 20.1 g, respectively. Serum, epididymal white adipose tissue, and gastrocnemius muscle were harvested at sacrifice for evaluations of cytokine concentration **(F)** and gene expression by qRT-PCR **(G, H)** as described in *Methods*. Data are shown as dot plots with bars representing mean ± SEM. *p < 0.05,**p < 0.01 and ****p < 0.0001 based on two-tailed Student’s t-test **(A–D)**, two-way ANOVA with Tukey’s multiple comparison test **(E, G, H)**, or use of a ROUT method (Q = 0.01) to remove outliers followed by Mann-Whitney test with Bonferroni multiple comparison test **(F)** for differences between cohorts injected with PBS alone to cohorts injected with PBS in the presence of RM9 cells.

Having established *in vitro* that 1) JAK inhibition of lipolysis was specific to signals from IL-6 family cytokines and 2) propranolol inhibition of lipolysis was specific to signals from β-adrenergic agonists, we next tested the potential of these forms of inhibition to block *in vivo* adipose wasting in a cancer cachexia animal model. We have previously demonstrated that the JAK inhibitors ruxolitinib and tofacitinib suppress adipose wasting by blocking systemic and local levels of IL-6 family cytokines in the allotransplanted C26c20 colon adenocarcinoma *in vivo* cachexia model, which is syngeneic to Balb/c mice (15). To generalize the potential importance of IL-6 family cytokines to the etiology of cachexia in other murine tumor models syngeneic to different host backgrounds, we next evaluated the allotransplanted RM9 prostate cancer *in vivo* cachexia model which is syngeneic to C57BL/6J mice. Compared to vehicle-treated animals (PBS), RM9 tumor-bearing mice developed significant tumor burden within 2 weeks of cancer cell allotransplant (**Figure 3A**). At sacrifice the RM9 tumor-bearing mice lost more than 10% of their body weight (**Figure 3B**), 50% of their fat mass (**Figure 3C**), and ~10% of their lean muscle mass (**Figure 3D**), and these mice displayed reduced food intake after the development of palpable tumor (**Figure 3E**, *days 8-15*).

The RM9-tumor bearing mice, compared to vehicle-treated animals, also had significant increases in systemic levels of multiple inflammatory cytokines that were either IL-6 family members — IL-6, LIF — or other cytokines such as IL-10 and G-CSF that can also increase JAK/STAT3 signaling in target tissues (**Figure 3F**) (25–27). The absolute serum concentrations of IL-6 and LIF observed during RM9-associated cancer cachexia were 83 ± 20 ng/mL and 3.4 ± 0.8 ng/ml, respectively. These concentrations are similar to those of recombinant IL-6 (3 nM = 65 ng/mL) and recombinant LIF (0.3 nM = 5.9 ng/mL) that induced STAT3 phosphorylation with subsequent glycerol and NEFA release in differentiated adipocytes (see **Figures 1A, B**). In parallel, we measured the mRNA expression of epididymal white adipose tissue and gastrocnemius muscle to evaluate the peripheral host tissue response during RM9 cancer cachexia. As we previously showed in a murine colon cancer cachexia model, there was an induction of *IL6* and *Socs3* mRNA expression with a decrease in *Lep* mRNA expression in epididymal white adipose tissue (**Figure 3G**) (15). Although *IL-6* and *Socs3* mRNA expression did not significantly change in the gastrocnemius muscle of RM9-bearing mice, *IL6ra* was increased similar to what others have observed in a murine pancreatic cancer cachexia model (**Figure 3H**) (28).

**Figure 4:**
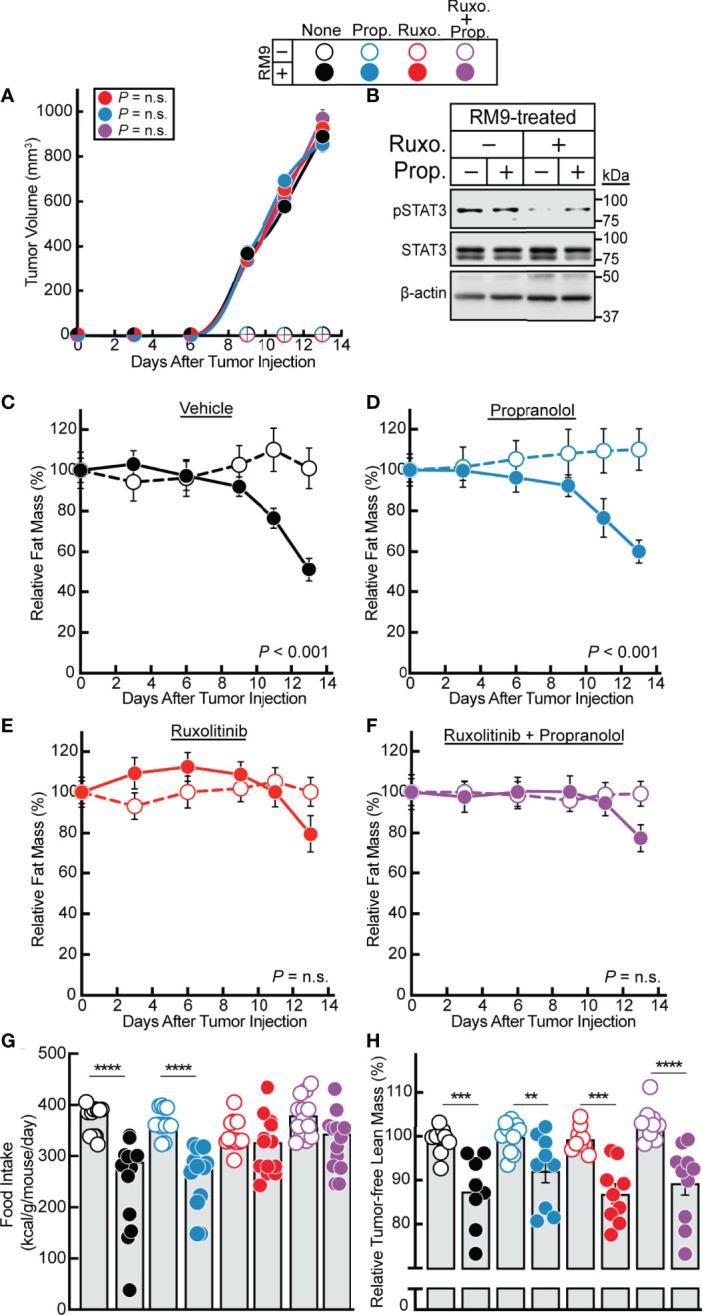
Effect of JAK and β-adrenergic Inhibitors on RM9 Cancer Cachexia Adipose Wasting. **(A–H)** Chow-fed C57BL/6 J mice (12-week old male mice, 4 mice/cohort) were injected s.c. in their right flank with PBS in the absence (*open circles*) or presence (*closed circles)* of 1x10^6^ RM9 cells on day 0 followed by i.p. administration of 150 µl PBS containing 2% DMSO (v/v) and 30% PEG300 (v/v) in the absence (*black*) or presence of 10 mg/kg propranolol (blue, *Prop.*), 50 mg/kg ruxolitinib (*red, Ruxo.*), or a combination of both compounds (*purple, Ruxo. + Prop.*) twice daily. Tumor volume **(A)**, immunoblot analysis of harvested white adipose tissue with the indicated antibodies **(B)**, and fat mass **(C–F)**, food intake **(G)**, and tumor-free lean mass **(H)** were measured at the indicated time points **(A, C–F)** or at sacrifice **(B, G, H)** as described in *Methods*. Fat and lean mass are shown relative to the average day 0 reference value for each respective cohort. The average fat mass values at day 0 for the mouse cohorts without RM9 cells were 3.1 g, 3.2 g, 3.0 g, and 3.0 g and with RM9 cells were 3.1 g, 3.3 g, 3.1 g, and 3.2 g for vehicle, propranolol, ruxolitinib, and both ruxolitinib and propranolol conditions, respectively. The average lean mass values at day 0 for the mouse cohorts without RM9 cells were 20.5 g, 20.2 g, 20.2 g, and 22.8 g and with RM9 cells were 20.2 g, 21.7 g, 21.1 g, and 21.8 g for vehicle, propranolol, ruxolitinib, and both ruxolitinib and propranolol conditions, respectively. Data are shown as mean ± SEM **(A, C–F)** or dot plots with bars representing mean ± SEM **(B, G, H)**. **p < 0.01, ***p < 0.01, and ****p < 0.001 or P value calculated by using a two-way repeated measures ANOVA **(A, C–F)** or by two-way ANOVA followed by Sidak’s multiple comparison test **(G, H)** to compare RM9 cohorts in the absence or presence of treatment **(A)** or matched PBS- and RM9-treated cohorts **(C–H)**. Not significant (n.s.).

Having established a strong association between STAT3-dependent cachexia-inducing cytokines in the RM9 system, we subsequently evaluated the effect of ruxolitinib and/or propranolol treatment in the RM9 *in vivo* cachexia model. We treated the RM9-tumor bearing mice with vehicle, propranolol, ruxolitinib, or the combination of propranolol and ruxolitinib and conducted longitudinal measurements of tumor size and adipose mass. We showed that tumor growth kinetics over time were not influenced by any pharmacologic intervention **(** **Figure 4A****)**. Verifying findings from our *in vitro* assays, ruxolitinib but not propranolol suppressed RM9 tumor-induced STAT3 phosphorylation in white adipose tissue (**Figure 4B**). Compared to vehicle-treated animals administered no tumor, those mice that were allotransplanted with RM9 cells leading to tumor formation displayed an ~50% reduction in fat mass over time (**Figure 4C**). Propranolol-treated RM9 cohorts also demonstrated a similar ~50% reduction in fat mass over time compared to control cohorts (**Figure 4D**). Only RM9 tumor cohorts treated with ruxolitinib demonstrated a suppression of cachexia-associated fat mass loss over time (**Figures 4E, F**). Ruxolitinib also suppressed the cachexia-associated anorexia phenotype observed in RM9 mice **(** **Figure 4G**) but did not block the lean mass loss associated with RM9-bearing mice (**Figure 4H**). The selective effect of ruxolitinib, but not propranolol, in suppressing adipose wasting in this cancer cachexia mouse model mirrored the selective effect of JAK inhibition on lipolysis that we previously observed with differentiated adipocytes. Taken together, these *in vitro* and *in vivo* findings further emphasize the role that cytokine-mediated JAK signaling plays in the induction of adipocyte lipolysis and adipose wasting in multiple murine models of cancer cachexia.

### IL-6- but Not β-Adrenergic-Mediated Adipocyte Lipolysis Requires STAT3

Previously, we demonstrated that JAK/STAT3 signaling is required for IL-6 family cytokine-induced adipocyte lipolysis by creating primary differentiated adipocytes from the adipose stromal vascular fraction of adipocyte-specific (Adiponectin-Cre) STAT3 knockout mice (7). Use of these differentiated adipocytes blocked IL-6 family of cytokine- but not isoproterenol-induced lipolysis. In this model, STAT3 is only knocked out in the adipocytes that fully differentiate and express adiponectin. Since the pre-adipocytes in this model still express STAT3 and the rate of differentiation in this model is 70-80%, there was still cells in the treated culture that expressed STAT3.

**Figure 5:**
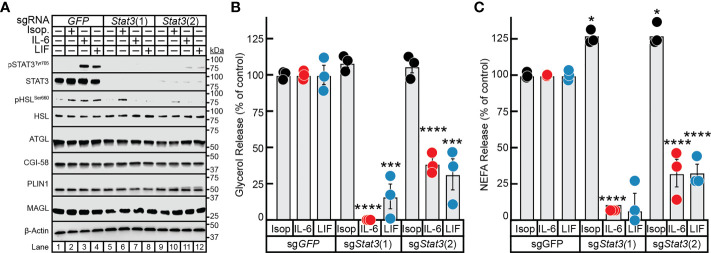
The Role of STAT3 in IL-6 and β-adrenergic Adipocyte Signaling and Lipolysis. **(A–C)** Pre-adipocytes in a 12-well format were infected with lentivirus containing Cas9 and the indicated sgRNA as described in *Methods*. On day 8, differentiated adipocytes were treated in a final volume of 1 ml of medium D with 3 µl DMSO and 3 µl PBS containing 300 nM isoproterenol, 3 nM IL-6, or 0.3nM LIF. After incubation for 24 hours, medium was collected and processed to measure concentrations of glycerol **(B)** or NEFA **(C)** and adipocytes were harvested and processed for immunoblot analysis **(A)** with the indicated antibodies as described in *Methods*. Glycerol release is shown relative to the respective stimulant in the sg*GFP* condition which were 366 μM, 70 μM, and 33 μM for isoproterenol, IL-6, and LIF, respectively. NEFA release is shown relative to the respective stimulant in the sg*GFP* condition which were 273 μM, 137 μM, and 34 μM for isoproterenol, IL-6, and LIF, respectively. Data is shown as mean ± SEM of three wells. *p < 0.5, ***p < 0.001, or ****p < 0.0001 based on two-way ANOVA with Tukey’s multiple comparison test for differences in relative glycerol or NEFA release between sgRNA treatments with the same stimulant. These results were confirmed in at least three independent experiments.

To overcome the deficiency in this model and to ensure that STAT3 expression was silenced not only in differentiated adipocyte but also in the minority of undifferentiated pre-adipocytes as well, we used CRISPR/Cas9 tools on the pre-adipocytes prior to terminal differentiation of these cells into mature adipocytes. Specifically, pre-adipocytes were treated with lentiviral-based CRISPR/Cas9 constructs targeting either *GFP* or two different regions of the *Stat3* gene–sg*Stat3(1)* and sg*Stat3(2)*—followed by differentiation of these cells into mature adipocytes. Immunoblot analysis was used to confirm near total suppression of STAT3 protein expression and cytokine-induction of STAT3 phosphorylation using sg*Stat3(1) (lanes 5-8)* and sg*Stat3(2)* (*lanes 9-12)* compared to control cells treated with sg*GFP* (*lanes 1-4*). (**Figure 5A**). Independent silencing of *Stat3* had no effect on the expression of other lipolysis-related molecules including ATGL, CGI-58, HSL, MAGL, and PLIN1 when compared to control conditions.

Isoproterenol induction of adipocyte lipolysis, as determined by glycerol release (**Figure 5B****)** and NEFA release (**Figure 5C****),** was not affected by silencing of *GFP* or by the two approaches of silencing *STAT3*. Conversely, silencing of *Stat3* with sg*Stat3(1)* led to near total suppression of glycerol release (**Figure 5B**) and complete suppression of NEFA release (**Figure 5C**) induced by IL-6 or LIF, in concordance with complete suppression of STAT3 protein expression (see **Figure 5A**, lanes 5-8). Additionally, cytokine-mediated glycerol and NEFA release were reduced to 75% of control levels in adipocytes treated with sg*Stat3(2)*, in concordance with the reduced efficiency of *Stat3* silencing by this sgRNA (**Figure 5A**, *lanes 9-12*). This correlation of *Stat3* silencing efficiency with selective inhibition of cytokine lipolysis across two distinct *Stat3*-targeting sgRNA sequences validates the specificity of lentiviral-based CRISPR/Cas9 approach in the adipocyte system. These findings highlight the requirement of STAT3 activation in cancer cachexia cytokine-mediated of adipocyte lipolysis and provide a new tool to more efficiently evaluate other molecules involved in cachexia-associated adipose wasting.

### ATGL but Not HSL Is Required for IL-6 Family Cytokine-Mediated Adipocyte Lipolysis

**Figure 6:**
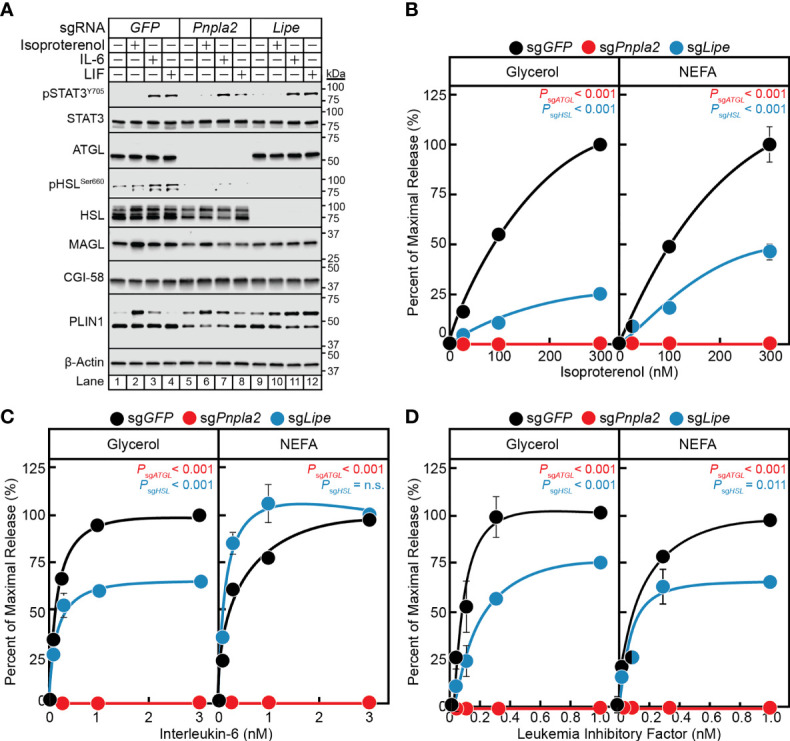
The Role of ATGL and HSL in IL-6 and β-adrenergic Adipocyte Signaling and Lipolysis. **(A–D)** Pre-adipocytes in a 12-well format were infected with lentivirus containing Cas9 and sg*GFP* (*black*), sg*Pnpla2* (*red*), or sg*Lipe* (*blue*) followed by differentiation as described in *Methods*. On day 8, differentiated adipocytes were treated in a final volume of 1 ml of medium D with 3 µl DMSO and 3 µl PBS containing the indicated concentration of isoproterenol **(A, B)**, IL-6 **(A, C)**, or LIF **(A, D)**. After incubation for 24 hours, medium was collected and processed to measure concentrations of glycerol (*left panel*) or NEFA (*right panel*) and adipocytes were harvested and processed for immunoblot analysis **(A)** with the indicated antibodies as described in *Methods*. Glycerol levels are shown relative to the average 300 nM isoproterenol condition (**B**, 578 µM), 3 nM IL-6 condition (**C**, 140 µM), or 1 nM LIF condition (**D**, 86 µM) of the sgGFP-treated adipocytes. NEFA levels are shown relative to the average 300 nM isoproterenol condition (**B**, 123 µM), 3 nM IL-6 condition (**C**, 57 µM), or 1 nM LIF condition (**D**, 27 µM) of the sg*GFP*-treated adipocytes. Data is shown as mean ± SEM of three wells. *P* value calculated by using a two-way repeated measures ANOVA between sg*GFP* and the indicated sgRNA conditions. These results were confirmed in at least three independent experiments. *Not significant (n.s.)*.

Having established a correlation between the *in vitro* cytokine-mediated adipocyte lipolysis assay and *in vivo* cancer cachexia models and validated a lentiviral-CRISPR/Cas9 knockout system in adipocytes, we next leveraged this *in vitro* assay to further characterize the steps contributing to cancer cachexia-associated adipocyte lipolysis. While the mechanism of isoproterenol-induced lipolysis through the actions of HSL is well characterized (29), the exact lipases that support IL-6 family cytokine induction of adipocyte lipolysis remain unknown. Therefore, we next compared the ability of IL-6, LIF, and isoproterenol to induce lipolysis in adipocytes lacking ATGL or HSL. Specifically, pre-adipocytes were treated with lentiviral-based CRISPR/Cas9 constructs targeting either *GFP*, *Pnpla2* (protein: ATGL) or *Lipe* (protein: HSL) followed by differentiation of these cells into mature adipocytes. Similar to the silencing of *Stat3*, we identified multiple sgRNAs that could silence the expression of *Pnpla2* or *Lipe* in our studies (data not shown), but we reported findings from use of a representative sgRNA for each of these genes in **Figure 6**. Immunoblot analysis was used to confirm complete suppression of ATGL *(lanes 5-8)* or HSL *(lanes 9-12)* protein expression compared to control cells with silencing of only *GFP* (*lanes 1-4*) (**Figure 6A**). Independent silencing of *Pnpla2* (protein: ATGL) had no effect on the expression of total HSL, CGI-58, MAGL, and PLIN1 when compared to control conditions. Cytokine-mediated phosphorylation of STAT3 also remained intact in the absence of ATGL protein expression. Silencing of *Lipe* (protein: HSL) had no effect on the expression of ATGL, CGI-58, MAGL, PLIN1, and cytokine-mediated STAT3 phosphorylation when compared to control conditions.

When measuring lipolysis potential, even at the highest concentrations of isoproterenol (**Figure 6B**), IL-6 (**Figure 6C**), and LIF (**Figure 6D**), blocking of ATGL expression with silencing of *Pnpla2* led to a complete suppression of NEFA and glycerol release across all conditions. Blocking HSL expression with silencing of *Lipe* in isoproterenol-treated adipocytes led to a ~75% reduction in glycerol release (*left panel*) and ~50% reduction in NEFA release (*right panel*) over increasing concentrations of isoproterenol (**Figure 6B**). Blocking HSL expression with silencing of *Lipe* in IL-6-treated adipocytes led to a significant reduction in glycerol release of ~40%, but did not show an effect on NEFA release (**Figure 6C**). Silencing of HSL expression in LIF-treated adipocytes led to a significant reduction in glycerol and NEFA release of ~25-35% (**Figure 6D**). Collectively, this data suggests that ATGL is required for both β-adrenergic- and IL-6 family cytokine-mediated adipocyte lipolysis. Compared to isoproterenol, however, IL-6 family of cytokines are significantly less reliant on HSL to induce adipocyte lipolysis. Both findings are congruent with the requirements of ATGL and to a lesser extent HSL for adipose wasting in murine models of cancer cachexia in *Pnpla*2^-/-^ or *Lipe*^-/-^ genetic backgrounds (30).

### CGI-58 but Not MAGL Is Required for IL-6 Family Cytokine-Mediated Adipocyte Lipolysis

**Figure 7:**
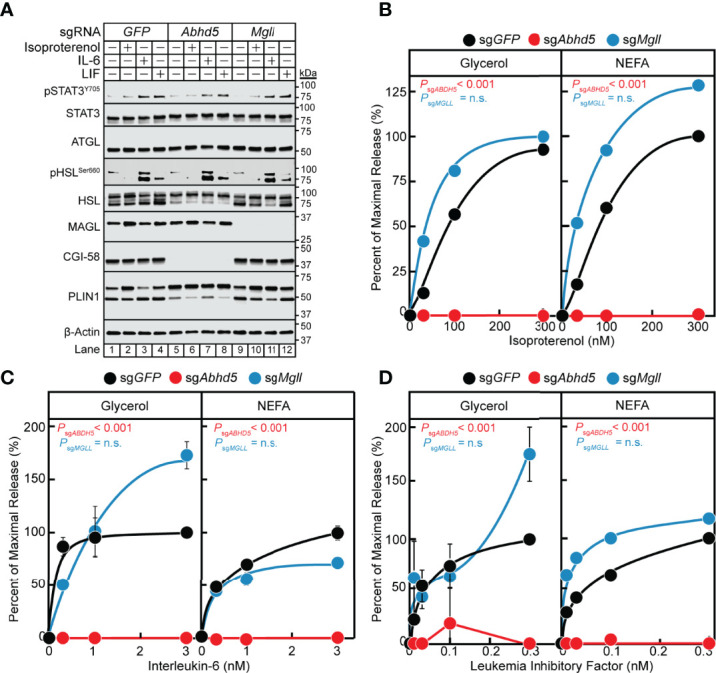
The Role of MAGL and CGI-58 in IL-6 and β-adrenergic Adipocyte Signaling and Lipolysis. **(A-D)** Pre-adipocytes in a 12-well format were infected with lentivirus containing Cas9 and sg*GFP* (*black*), sg*Abhd5* (*red*), or sg*Mgll* (*blue*) followed by differentiation as described in *Methods*. On day 8, differentiated adipocytes were treated in a final volume of 1 ml of medium D with 3 µl DMSO and 3 µl PBS containing the indicated concentration of isoproterenol **(A, B)**, IL-6 **(A, C)**, or LIF **(A, D)**. After incubation for 24 hours, medium was collected and processed to measure concentrations of glycerol (*left panel*) or NEFA (*right panel*) and adipocytes were harvested and processed for immunoblot analysis **(A)** with the indicated antibodies as described in *Methods*. Glycerol levels are shown relative to the average 300 nM isoproterenol condition (**B**, 653 µM), 3 nM IL-6 condition (**C**, 109 µM), or 0.3 nM LIF condition (**D**, 55 µM) of the sgGFP-treated adipocytes. NEFA levels are shown relative to the average 300 nM isoproterenol condition (**B**, 282 µM), 3 nM IL-6 condition (**C**, 70 µM), or 1 nM LIF condition (**D**, 21 µM) of the sg*GFP*-treated adipocytes. Data is shown as mean ± SEM of three wells. *P* value calculated by using a two-way repeated measures ANOVA between sg*GFP* and the indicated sgRNA conditions. These results were confirmed in at least three independent experiments. *Not significant (n.s.)*.

In the previous section, we established that the IL-6 family of cytokines require the lipase ATGL to transduce their adipocyte lipolysis signals. We next evaluated the influence of other lipolysis-related molecules on cytokine-mediated lipolysis, including ATGL co-activator CGI-58/ABHD5 (gene: *Abhd5*) and lipase MAGL (gene: *Mgll*). We measured IL-6, LIF, and isoproterenol lipolysis induction in adipocytes absent CGI-58 or MAGL protein expression through use of lentiviral CRISPR/Cas9 gene-silencing techniques in pre-adipocytes followed by terminal differentiation into mature adipocytes. Similar to the silencing of *Stat3*, we identified multiple sgRNAs that could silence the expression of *Abhd5* and *Mgll* in our studies (data not shown), but we reported findings from use of a representative sgRNA for each of these genes in **Figure 7**. Immunoblot analysis was performed on the same set of treated adipocytes for STAT signaling and expression of lipolysis-associated molecules (**Figure 7A**). Independent silencing of *GFP*, *Abhd5* (protein: CGI-58), or *Mgll* (protein: MAGL) only led to decreased expression of the intended target and had no effect on the expression of other lipolysis-related molecules or extent of cytokine-induced STAT3 phosphorylation. Across increasing concentrations of isoproterenol (**Figure 7B**), IL-6 (**Figure 7C**), and LIF (**Figure 7D**), blocking CGI-58 protein expression led to a complete suppression of NEFA and glycerol release under all conditions. Blocking MAGL protein expression did not significantly reduce isoproterenol- or IL-6 family cytokine-stimulated glycerol or NEFA across increasing concentrations of each respective stimulus (**Figures 7B–D**). Collectively, these data suggest that the coactivator of ATGL, CGI-58, is also required for cancer cachexia-associated IL-6 family cytokine- and β-adrenergic-mediated adipocyte lipolysis

## Discussion

Despite representing phenotypic extremes of adiposity, both cancer cachexia and obesity are disease states associated with adipose inflammation, increases in local and circulating levels of IL-6 family cytokines (e.g. IL-6 and LIF), and elevated adipocyte lipolysis (13, 14, 31, 32). Although inflammation and lipolysis are independently associated with both pathologic conditions, there is limited mechanistic insight into how inflammatory cytokines induce adipocyte lipolysis. To elucidate the impact of adipose inflammation on the adipocyte, we compared the well-studied β-adrenergic agonist isoproterenol to IL-6 family cytokines in their ability to signal lipolysis in differentiated adipocytes. Signaling by β-adrenergic agonists are also associated with cancer cachexia, providing important justification for a comparison of its lipolytic action to that of inflammatory cytokines. At the upstream signaling level, we determined that β-adrenergic agonists and IL-6 family cytokines had divergent mechanisms of action. Where isoproterenol required intact β-adrenergic signaling to induce lipolysis rapidly, IL-6 family cytokines required an intact JAK/STAT3 signaling axis that supported increasing adipocyte lipolysis over a more delayed time frame. Cycloheximide, the pan-translation inhibitor, suppressed cytokine- but not isoproterenol-induced lipolysis, verifying that the delayed lipolysis induced by this class of cytokines is likely due to a reliance on *de novo* transcription/translation. JAK inhibitors, but not the β-adrenergic antagonist propranolol, correspondingly suppressed STAT3 phosphorylation in white adipose tissue while blocking adipose wasting in cancer cachexia murine models. Finally, use of CRISPR/Cas9 techniques in pre-adipocytes confirmed the requirement of STAT3 in transmitting cachexia-associated, cytokine-induced lipolysis signals and validated this tool to further elucidate other molecules necessary for permitting cachexia-associated adipose wasting. Ultimately, we demonstrated that the lipase ATGL and its cofactor CGI-58 are also required for this lipolysis, while the lipases HSL and MAGL are not. Our findings collectively define how cytokines contribute directly to cachexia-associated adipose wasting through STAT3-dependent transcriptional changes that ultimately increase adipocyte lipolysis through ATGL and CGI-58 activity, providing new potential targets to suppress cancer cachexia.

Use of the *in vitro* adipocyte assay to compare isoproterenol- and IL-6 family cytokine-mediated lipolysis highlighted divergent actions between these lipolytic stimuli. Whereas isoproterenol uses β-adrenergic signaling and induces lipolysis rapidly, cancer cachexia-associated cytokines use JAK/STAT3 signaling to transcriptionally reprogram the adipocyte to increase lipolysis over time in an ATGL/CGI-58 dependent manner. This same *in vitro* assay was previously used to screen multiple JAK inhibitors that blocked IL-6 family cytokine-induced lipolysis, correlating with *in vivo* suppression of adipose wasting in the C26c20 murine cancer cachexia (Balb/c) model. We were able to generalize these findings in the current study, showing that the same JAK inhibitor and not the β-adrenergic inhibitor propranolol could suppress cancer cachexia-associated adipose wasting in a different primary tumor murine cancer cachexia model (RM9) that is syngeneic to another genetic background (C57BL/6J). The effectiveness of the JAK inhibitors in the RM9 cancer cachexia model was most likely due to the elevation of IL-6 family of cytokines as well as other STAT3-activating cytokines (see **Figure 3F**). These findings suggest that targeting of only one cytokine may be ineffective in suppressing cancer cachexia development, explaining why anti-TNFα and anti-IL-6 clinical trials failed to show a benefit with respect to tissue preservation and survival (33). It is likely that targeting of signaling pathways common to multiple cachexia-associated cytokines may offer the most durable treatment approach for cancer cachexia. In light of these preclinical findings, we have been approved to activate an early phase clinical trial assessing the safety of using the JAK inhibitor ruxolitinib in advanced non-small cell lung cancer patients with cancer cachexia (ClinicalTrials.gov Identifier: NCT04906746).

Our *in vitro* studies shed additional light on how IL-6 family cytokines and isoproterenol induce adipocyte lipolysis downstream of STAT3-dependent transcriptional activity. Genetically silencing multiple lipolysis molecules from adipocytes identified that ATGL and its cofactor CGI-58 are required to transduce the cachexia-associated cytokine signaling of increased adipocyte lipolysis. In contrast, the lipases HSL was not required. This *in vitro* data is consistent with the *in vivo* data of Zechner and colleagues which demonstrated that global ATGL knockout mice were resistant to cancer cachexia-associated adipose and skeletal mass loss (30). Furthermore, this study showed that HSL knockout mice were still susceptible to cancer cachexia-associated adipose and muscle wasting. The therapeutic window of ATGL inhibitors could be narrow due to the fact ATGL knockout mice show cardiac steatosis and cardiomyopathy (34). Therefore, identification of other molecules necessary for cytokine-induced adipocyte lipolysis could provide new targets to block cancer cachexia *in vivo* and in clinical trials. To this end, we further characterized the role of other lipases and cofactors that are responsible for IL-6 family of cytokine-induced lipolysis and identified that the ATGL cofactor CGI-58 is also required, whereas the acyl glycerol lipase MAGL is not. Our data highlights the potential use of CGI-58 as a target to limit cancer cachexia development. Though the protein levels of ATGL and CGI-58 are not altered during STAT3 activation of lipolysis, we are currently evaluating cytokine effects on their temporal spatial interactions on lipid droplets during lipolysis as previously described with isoproterenol (35). We also plan to evaluate the role of CGI-58 in modulating cancer cachexia with adipose-specific knockouts of the respective gene in conditions of tumor-associated wasting.

The *in vitro* adipocyte inflammation/lipolysis assay appears to be an accurate surrogate for *in vivo* cachexia models permitting 1) the identification of pathways critical to cytokine-mediated adipocyte lipolysis that correlate with *in vivo* cachexia-associated adipose wasting and 2) the screening of compounds including specific JAK inhibitors that suppress this cytokine-mediated adipocyte lipolysis and cachexia-associated adipose wasting. The significant correlation in findings between our *in vitro* adipocyte inflammation assay and *in vivo* cancer cachexia models point to the utility of the *in vitro* assays in offering an early insight into the biology of cytokine-induced adipocyte lipolysis relevant to tumor-host interactions in cancer cachexia itself and as a screening tool of cancer cachexia lipolysis regulators. To this end, we are presently using an unbiased RNA sequencing-based approach to identify differentially expressed genes that are uniquely altered during cytokine-mediated reprogramming of adipocytes to amplify the inflammation signal and to identify new targets of cachexia-associated cytokine adipocyte lipolysis. Furthermore, the successful and complete silencing of molecules using the lentiviral-CRISPR/Cas9 approach in pre-adipocytes will allow for efficient validation of lipolysis-related signaling pathways in cancer cachexia.

In summary, our studies have provided insight into the mechanisms underlying adipocyte lipolysis induction by IL-6 family cytokines in models replicating cancer cachexia-associated adipose wasting using an *in vitro* adipocyte lipolysis assay and *in vivo* murine cancer cachexia models. Unlike classically described lipolytic agents including isoproterenol, the IL-6 family cytokines contribute to adipocyte lipolysis though JAK-dependent STAT3 activation with time-dependent downstream *de novo* gene expression changes. Furthermore, our data demonstrates that IL-6 family cytokines are reliant on ATGL and CGI-58 activity to promote lipolysis. We have ultimately leveraged our novel *in vitro* adipocyte inflammation assay and its accurate surrogacy of cancer cachexia murine models to prove that IL-6 family cytokines and their induction of JAK/STAT3 signaling are relevant to multiple primary cancer types that induce cachexia-associated adipose wasting, paving the way for the initiation of an early phase clinical trial. Identifying and characterizing other molecules intrinsic to cancer cachexia-associated cytokine signal transduction that lead to adipocyte lipolysis could provide novel targets for cancer patients with cachexia.

## Data Availability Statement

The raw data supporting the conclusions of this article will be made available by the authors, without undue reservation.

## Ethics Statement

The animal study was reviewed and approved by University of Texas Southwestern Medical Center Institutional Animal Care and Use Committee.

## Author Contributions

AYG, JY, AG, TG, PI, and RI designed experiments and analyzed data. AYG, JY, AG, and TG conducted experiments. AYG performed statistical analysis. AYG, RI, and PI wrote the manuscript. All authors contributed to the article and approved the submitted version.

## Funding

This work was supported by the Burroughs Wellcome Fund Career Awards for Medical Scientists (1019692), American Cancer Society grant (133889-RSG-19-195-01-TBE), American Gastroenterological Association Research Scholar Award (R2019AGARSA3), Cancer Prevention and Research Institute of Texas (RP200170), V Foundation V Scholar Program (V2019-014), and National Institute of Health (1RO1DK128166-01A1, 5P01-HL20948, P30CA142543, and 5T32GM007062-44).

## Conflict of Interest

The authors declare that the research was conducted in the absence of any commercial or financial relationships that could be construed as a potential conflict of interest.

Pfizer, Inc. is currently supporting a collaborative project with the REI laboratory that is independent of all data presented in this manuscript.

## Publisher’s Note

All claims expressed in this article are solely those of the authors and do not necessarily represent those of their affiliated organizations, or those of the publisher, the editors and the reviewers. Any product that may be evaluated in this article, or claim that may be made by its manufacturer, is not guaranteed or endorsed by the publisher.

## Abbreviations

Comparative Gene Identification 58 (gene: *Abhd5*, protein: CGI-58), adipose triglyceride lipase (gene: *Pnpla2*, protein: ATGL), hormone sensitive lipase (gene: *Lipe*, protein: HSL), interleukin-6 (IL-6), isoproterenol, Janus kinase (JAK), leukemia inhibitory factor (LIF), monoacylglycerol lipase (gene: *Mgll*, protein: MAGL), non-esterified fatty acid (NEFA), signal transducer and activator of transcription 3 (STAT3), triacylglycerol (TAG).
